# DES-ROD: Exploring Literature to Develop New Links between RNA Oxidation and Human Diseases

**DOI:** 10.1155/2020/5904315

**Published:** 2020-03-27

**Authors:** Magbubah Essack, Adil Salhi, Christophe Van Neste, Arwa Bin Raies, Faroug Tifratene, Mahmut Uludag, Arnaud Hungler, Bozidarka Zaric, Sonja Zafirovic, Takashi Gojobori, Esma Isenovic, Vladan P. Bajic

**Affiliations:** ^1^Computer, Electrical and Mathematical Sciences and Engineering Division (CEMSE), Computational Bioscience Research Center, Computer (CBRC), King Abdullah University of Science and Technology (KAUST), Thuwal 23955-6900, Saudi Arabia; ^2^Laboratory of Radiobiology and Molecular Genetics, Institute of Nuclear Sciences Vinca, University of Belgrade, Mike Petrovica Alasa 12-14, 11000 Belgrade, Serbia; ^3^Biological and Environmental Sciences and Engineering Division (BESE), King Abdullah University of Science and Technology (KAUST), Thuwal 23955-6900, Saudi Arabia

## Abstract

Normal cellular physiology and biochemical processes require undamaged RNA molecules. However, RNAs are frequently subjected to oxidative damage. Overproduction of reactive oxygen species (ROS) leads to RNA oxidation and disturbs redox (oxidation-reduction reaction) homeostasis. When oxidation damage affects RNA carrying protein-coding information, this may result in the synthesis of aberrant proteins as well as a lower efficiency of translation. Both of these, as well as imbalanced redox homeostasis, may lead to numerous human diseases. The number of studies on the effects of RNA oxidative damage in mammals is increasing by year due to the understanding that this oxidation fundamentally leads to numerous human diseases. To enable researchers in this field to explore information relevant to RNA oxidation and effects on human diseases, we developed DES-ROD, an online knowledgebase that contains processed information from 298,603 relevant documents that consist of PubMed abstracts and PubMed Central full-text articles. The system utilizes concepts/terms from 38 curated thematic dictionaries mapped to the analyzed documents. Researchers can explore enriched concepts, as well as enriched pairs of putatively associated concepts. In this way, one can explore mutual relationships between any combinations of two concepts from used dictionaries. Dictionaries cover a wide range of biomedical topics, such as human genes and proteins, pathways, Gene Ontology categories, mutations, noncoding RNAs, enzymes, toxins, metabolites, and diseases. This makes insights into different facets of the effects of RNA oxidation and the control of this process possible. The usefulness of the DES-ROD system is demonstrated by case studies on some known information, as well as potentially novel information involving RNA oxidation and diseases. DES-ROD is the first knowledgebase based on text and data mining that focused on the exploration of RNA oxidation and human diseases.

## 1. Background

Oxidative damage induced by reactive oxygen species (ROS) to the cellular elements such as proteins, lipids, and DNA has proven to be deleterious to organisms as a whole. Until recently, RNA damage was not recognized and explored as one such component of ROS effects on cellular elements. RNA oxidation was believed to be primarily a consequence of a dying cell until it was shown that changes in RNA structure are early events in the development of aging-related disorders such as Alzheimer's disease (AD), Parkinson's disease (PD), amyotrophic lateral sclerosis (ALS), and cardiovascular diseases (CVD) [1–6]. These findings are strengthened by the notion that RNA species rRNA and tRNA are abundantly present in the cell and are not readily degraded during cell growth. These features have recognized RNA oxidation to be a great challenge to cell function and to cell surveillance mechanisms that control oxidative-reductive stress. Alterations of these processes may advance the development of pathologies in various diseases [1, 6–8].

RNA undergoes oxidative damage more often than DNA, owing to the RNA's location in the cytosol, where they are in closer proximity to the mitochondria where oxidative stress is generated and because there are no protective histones in single-stranded RNA structure [1]. These oxidative modifications of RNA affect translation and synthesis of proteins, as well as the self-regulatory processes of transcription repression by various miRNAs [9, 10]. Thus, curbing the accumulation of oxidatively damaged RNA aids the maintenance of cellular health and prevents disease development. Several RNA oxidation surveillance mechanisms prevent such accumulation. That is, oxidized RNAs appear to be targeted for degradation [11] in a process that involves the ribosome [12, 13]. Another RNA oxidation surveillance mechanism is via RNA-binding proteins such as YB-1, which was shown to bind to 8-oxoguanosine (8-oxoG) with high affinity [14]. This action of YB-1 and its known role in mRNA stability associated with helping the winding of RNA duplexes suggest that this protein may be functioning as an RNA chaperone that targets oxidized RNA for degradation [14]. Another mechanism of RNA quality control is promoted by proteins such as MTH1, MTH2, and NUDT5. These proteins can hydrolyze oxidatively damaged RNA (such as 8-oxoG), thereby eliminating them from the RNA precursor pool [15]. Many such research findings showed the complexity and importance of the RNA oxidation-related processes. However, research related to RNA oxidation mechanisms and its role in different diseases is scattered in a large volume of scientific literature. For example, indexed in the Web of Science (All Databases) (https://clarivate.com/), specifically focused on the RNA oxidations in human diseases, there are 50,905 and 273,633 scientific articles published in 2018 and the 2014-2018 period, respectively, while in the most strict selection of the Web of Science Core Collection, there are 21,578 and 100,016 articles published in 2018 and the 2014-2018 period, respectively. This volume of literature makes it infeasible to efficiently search for RNA oxidation-related information or track significant developments manually. Such bottlenecks are not new to specialized domains; thus, several groups have been looking for ways to simplify the search for useful information.

## 2. Exploring Voluminous Information

It has been acknowledged that automated systems are needed to search for and retrieve useful information from such voluminous data. Thus, several automated systems have been developed using text mining (TM) and/or natural language processing (NLP) for over 30 years [16–23]. Moreover, TM and NLP methods have been combined with different approaches for knowledge extraction from free text. For example, ontologies provide a systematic representation of interrelationships between terms in a specific domain [24, 25]. Various ontology-based frameworks have been developed [26] such as Aber-OWL [27]. Additionally, ontology-based systems have different purposes, for example, identifying pathways using pharmacogenomics data [28] and selecting gene candidates [29]. Other methods are based on network analysis [30] and biological knowledge graphs [31]. In addition, TM has been combined with methods from bioinformatics. For example, position weight matrices have been used for text representation and feature generation in a TM system to extract associations between methylated genes and diseases [32, 33]. Another study combined TM and bioinformatics approaches for the interpretation of mutations in protein kinases [34].

Several generalized automated systems were designed to facilitate extracting information from biomedical literature [35]. For example, iHOP uses a text mining approach wherein genes and proteins are used as hyperlinks between sentences and PubMed abstracts and then uses the text-mined information to produce network representations that users can browse [36]. Other tools include Twister that is aimed at reducing the screening time of systematic literature reviews [37]; SWIFT-Review, which is a workbench for systematic review based on NLP [38]; SparkText, which is a big data framework for mining biomedical literature [39]; and GIS, which is an NLP-based framework for gene discovery from scientific literature [40]. In addition to these tools, several frameworks for mining biomedical literature have been developed [41–47].

Automated extraction of relevant and necessary information helps improve our understanding and knowledge of specific domains, propose hypotheses, and potentially discover new knowledge. For example, TM and NLP systems have been used to identify new candidate compounds for drug repurposing [48, 49], analyze relationships between proteostasis protein factors and cancer [50], prioritize cancer genes and pathways [51], predict protein functions [52], and extract disease-related biomarkers [53], as well as find associations between TFs [54]. Additionally, the text has been used as features to represent protein structures and subsequently predict their characteristics computationally [55]. Other useful applications of TM and NLP have been reported in the literature [56–61].

Moreover, various domain-specific knowledgebases (KB) exist. For example, CRAB is a KB implemented to support chemical health risk assessment through literature TM [61]. Another KB is ERIC, developed to support research focused on molecular mechanisms of bacterial enteropathogens using TM of PubMed abstracts [62]. Also, CNVdigest created using TM assists geneticists or physicians to find rare CNVs and the original literature context for more detailed information [63]. Other tools include CHAT (Cancer Hallmark Analytics Tool), developed to organize and evaluate cancer-related scientific literature [64]; FamPlex, designed for exploring associations between human protein families and complexes in the scientific literature [65]; and PPInterFinder and PIminer, implemented for mining protein-protein interactions from biomedical text [66, 67], while [54] presents a tool for context-specific protein interaction networks based on TM. In addition to these tools, several tools have been developed for specialized domains [68].

Here, we develop DES-ROD, the KB focused on RNA oxidation-related research, and demonstrate its utility in this domain, focusing on the role of RNA oxidation in the development of AD, CVD, and obesity.

## 3. The DES-ROD Exploration System

We developed DES-ROD using the DES V3.0 framework on 26 November 2018. DES is a text mining and data mining system that allows the exploration of text through enriched concepts and enriched pairs of concepts in topic-specific literature. We used the DES framework to create several topic-specific KBs [32, 33, 54, 67, 69–82]. The underlying systems, workflow, and concept enrichment process used in the current version of DES have been described in [69]. The user manual is provided at https://des-documentation.readthedocs.io/en/des-rod/.

Specific to this DES-ROD, our local MongoDB repository (updated September 03, 2018) hosting PubMed and PubMed Central articles was used to retrieve all topic-specific articles using the following query: “*(human OR mouse OR rat OR mammal*∗*) AND (“RNA damage” OR Fenton OR PNPase OR hPNPase OR APE1 OR “apyrmidinic endonuclease 1” OR “apurinic endonuclease 1” OR nucleophosmin*∗*OR NPM1 OR “purine nucleoside phosphorylase” OR PNP OR “oxidative demethylase” OR “tRNA nucleotidyl transferase” OR “Y box binding protein” OR “Ro autoantigen” OR “8-hydroxyguanine” OR “8-oxoG” OR “8-hydroxyguanosine” OR “8-oxo-deoxyguanosine triphosphate” OR “8-oxodGTP” OR “8-oxo-guanosine triphosphate” OR “8-oxo-GTP” OR “nucleoside-diphosphate kinase” OR NDK OR “adenosine-diphosphate kinase” OR ADK OR Lipoxygenase*∗*OR LOs OR “4-hydroxy-2,3-nonenal” OR HNE OR “4-oxo-2-nonenal” OR acrolein OR “reductive stress” OR radical*∗*OR peroxide*∗*OR ROS OR “reactive oxygen species” OR RNS OR “reactive nitrogen species” OR redox OR “reduction-oxidation reaction” OR oxidat*∗*OR nitrosat*∗*OR peroxide*∗*OR superoxide*∗*OR detoxifi*∗*OR antioxid*∗*OR “polyunsaturated fatty acids” OR “arachidonic acid” OR “linoleic acid” OR hydroperoxide*∗*OR “hypochlorous acid” OR peroxynitrit*∗*OR flavoprot*∗*OR oxidase*∗*OR “cytochromes P450” OR catalase*∗*OR sulfiredoxin*∗*OR peroxiredoxin*∗*) AND (clinic*∗*OR disease*∗*OR diabet*∗*OR obes*∗*OR syndrome*∗*OR neuro*∗*OR heart OR cardi*∗*OR cancer*∗). The query retrieved 286,370 articles used as the literature corpus. This literature corpus was indexed using 38 dictionaries: 28 dictionaries from the preexisting DES v2.0 vocabularies (used to develop other KBs) and 10 newly compiled topic-relevant dictionaries (see Table 1).

To integrate these newly compiled dictionaries into DES-ROD, redundant dictionary concepts are unified and concepts are normalized to ensure that a single concept represents synonymous symbols and names. Then, initial indexing is performed to identify and remove promiscuous or ambiguous concepts. After this dictionary cleaning, the literature corpus is reindexed to calculate and ensure the accuracy of concepts' enrichment estimates.

Concepts are recognized as enriched, if their occurrence in the DES-ROD literature corpus is proportionally higher than its occurrence in the complete set of PubMed and PubMed Central articles in our local repository and has a false discovery rate (FDR) < 0.05. A total of 131,741 concepts were determined to be statistically enriched in DES-ROD (see Table 1). Also, 10,846,802 pairs of concepts were determined to be statistically enriched. Concepts are regarded as cooccurring based on their cooccurrence in the text within a 200-character distance from each other. The resulting network of concept pairs was also embedded in a high-dimensional semantic space, enabling the computation of semantic similarity between concepts. The literature corpus, 38 dictionaries, enriched concepts, enriched pairs of concepts, and semantic similarities were integrated to create DES-ROD.

## 4. Knowledgebase Utilities

DES-ROD allows RNA oxidation-related literature to be easily explored using concepts found to be statistically enriched in the topic-specific literature. The KB is designed to provide users with multiple means to explore the literature with topic-relevant concepts (determined through concept enrichment estimates). Users are provided with multiple views, including “Enriched Concepts”, “Enriched Pairs”, “Semantic Similarity”, and “Literature”. Briefly, individual-enriched concepts can be explored on the “Enriched Concepts” page where their mentions in the text are highlighted on the right-hand side annotation pane, enriched cooccurring concepts on the “Enriched Pairs” page are also linked to their cooccurrence context in the literature, and concepts with semantic similarity to a chosen enriched concept are displayed on the “Semantic Similarity” page. The “Semantic Similarity” link is new in this version of DES. Using these utilities, users can view all enriched concepts, search for their concept of interest, or select a specific dictionary. Furthermore, provided is a “Column visibility” tab that allows viewing the enriched concepts using several ranking options, including false discovery rate (FDR), KB frequency, background frequency, or density. Moreover, highlighting the concept or concept pair of interest allows the user to view the literature from where the indexing was retrieved. Also, concepts are highlighted, making them easily identifiable in the literature, as well as color-coded to indicate in which dictionary the concept is located. Each concept is also linked to a right-click menu which allows users to generate a “Network” view or “Term Co-occurrences” table. The literature in DES-ROD can also be explored via the “Literature” view. Case study examples are given below to demonstrate the utility of DES-ROD.

## 5. Case Studies that Demonstrate the Use of DES-ROD as a Research Supporting System

**Example 1 .**

Hypothesis derived through the use of DES-ROD.

**Hypothesis:** *Let-7b may be preventing RNA oxidation through suppression of OGG1, and this may be the cause of dopaminergic neuron death and Alzheimer's disease.*

Only recently was it reported that ROS could oxidatively modify miRNAs. Wang et al. [107] demonstrated that oxidatively modified miR-184 associates with the 3′ UTRs of some mRNAs (Bcl-xL and Bcl-w that are known to initiate apoptosis) that are not the usual targets of this miRNA. In this manner, oxidized miR-184 promotes apoptosis via suppression of Bcl-xL and Bcl-w. Also, miR-205/let-7/miR-184 is highly expressed in the nondiseased brain, and miR-205 directly inhibits LRKK2 [108]. In line with this, dopamine neurons were shown to be devoid of LRRK2 mRNA [109]. Moreover, the other miRNAs, miR-184 and let-7, repress E2F1 and DP, respectively, and downregulation of E2F1 and DP suppresses the death of dopaminergic neurons [110]. Also, inhibition of both let-7 and miR-184 is sufficient to phenocopy pathogenic LRRK2 in wild-type animal models, and both miRNAs regulate dopaminergic survival and activity [110]. This finding is interesting as the death of dopaminergic neurons is being looked at as the possible leading cause of both Alzheimer's disease (AD) and Parkinson's diseases (PD), and oxidized miR-184 not binding to its usual mRNA targets suggests that oxidized miR-184 might not be providing protection against the death of dopaminergic neurons. This reveals the complexity and necessity of oxidation research.

Also, RNA oxidation was shown to be significantly elevated in early preclinical stages of AD, and this increase is observed with a compensatory increase in 8-oxoguanine glycosylase (OGG1) levels [111, 112]. OGG1 is the primary enzyme responsible for the excision of 8-oxoguanine (8-oxoG), a mutagenic base byproduct of reactive oxygen species (ROS) that may be responsible for the RNA oxidation. Knowing that a single miRNA can modulate multiple genes and that multiple miRNAs are usually involved in a single disease or physiological phenotype, discerning the overall intricacies of these complex networks is needed. Thus, we here use DES-ROD to explore miRNA associated with RNA oxidation in AD.

**Figure 1:**
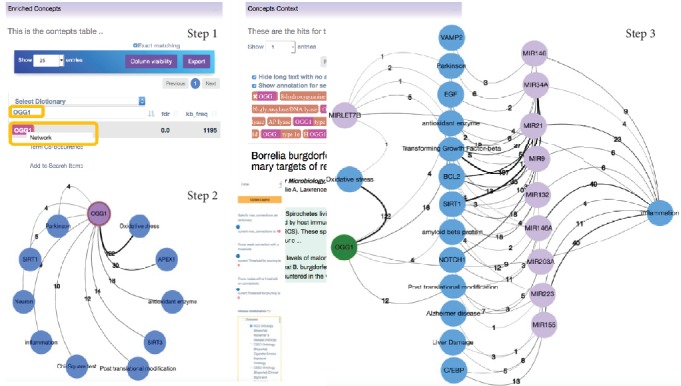
Step-by-step illustration of how DES-ROD can be used to identify relationships between the concepts. The blue circles represent nodes from the “ADO Ontology (BioPortal) Alzheimer's Disease Ontology” dictionary, the green circles represent the nodes from the “Human Genes and Proteins (EntrezGene)” dictionary, and the light purple circles represent the nodes from the “Human microRNAs” dictionary. The edge color is distributed across a color spectrum from black (strong association) to grey (weaker association) based on the frequency of cooccurrence. The number of publications that link the associated nodes is displayed on each edge. Note that the generated networks were exported from DES-ROD and manually adjusted in Cytoscape for better visibility.

In search of novel insights, we looked at AD concepts associated with OGG1. Thus, we explored DES-ROD by clicking on the “Enriched Concepts” link. In the search bar, we typed the concept of interest “OGG1” and then used the concepts' right-click menu to generate a network (Figure 1, Step 1). On the “Network” page, we selected the “ADO Ontology (BioPortal) Alzheimer's Disease Ontology” dictionary; then, the “OGG1” node was highlighted (Figure 1, Step 2) and expanded with the top ten enriched associated terms from the selected dictionary. This process was repeated by selecting the “Human microRNAs” dictionary only and then expanding the “inflammation” node with these concepts, as oxidative stress generally leads to inflammation. We then selected the “ADO Ontology (BioPortal) Alzheimer's Disease Ontology” dictionary only and then expanded all the microRNA nodes with concepts from this dictionary. All nodes with a single edge were removed and were nonspecific nodes such as “things related to severe stage”, “micro RNA”, “Chi-Square test”, “in vivo model”, and “In silico thing” (see Figure 1, Step 3).

Of the miRNAs retrieved, only “MIRLET7B” (referred to in the text as Let-7b) was associated with oxidative stress. Elevated levels of Let-7b have been detected in AD patients [113], and it was further identified as a blood-based molecular biomarker signature in AD [114]. However, we found no literature connecting Let-7b and OGG1 despite this indirect association depicted by the network generated by DES-ROD. Consequently, we used miRDB for microRNA target prediction [115]. This tool retrieved several predicted targets of “MIRLET7B” including OGG1. This finding indicates that Let-7b might have a direct role in RNA oxidation surveillance that protects against the development of AD.

**Example 2 .**

Finding the relevant concepts and potentially new knowledge derived through the use of DES-ROD: *focused on the association between type 2 diabetes and heart failure.*

Finding ROS-induced DNA damage in atherosclerosis led Martinet et al. [116] to assess whether oxidative stress-induced RNA damage occurs in human atherosclerotic plaques. They reported that 11 of 20 atherosclerotic plaques assessed showed significant loss of RNA integrity and strong staining for the oxidative damage marker 8-oxoG, compared to 20 nonatherosclerotic mammary arteries. Moreover, they showed that plaque pretreated with RNase A diminished in cytoplasmic 8-oxoG staining, which suggests RNA damage [116]. Also, in the mouse model of myocardial injury, oxidative modification of miR-184 results in decreased levels of Bcl-xL and Bcl-w, which are essential for apoptosis of cells [107]. On the other hand, different types of miRNA that are present in the cardiomyocytes, such as miR-1, miR-499, and miR-208, are not affected by RNA oxidation. This finding raises the possibility to suspect the presence of specific sequences that could be subjected to RNA oxidation and shows that RNA oxidation plays a role in the development of cardiovascular diseases.

Also, type 2 diabetes mellitus (T2DM) patients usually have high urinary levels of 8-oxo-7,8-dihydroguanosine (8-oxoGuo) and are at risk of cardiovascular mortality. Consequently, Kjaer et al. [117] set out to determine if 8-oxoGuo is associated with this cardiovascular mortality risk. They conducted a five-year follow-up clinical study on 1,863 patients with T2DM wherein they measured the level of 8-oxoGuo. It was concluded that in patients with type 2 diabetes, high RNA oxidation is associated with cardiovascular mortality risk [117].

**Figure 2:**
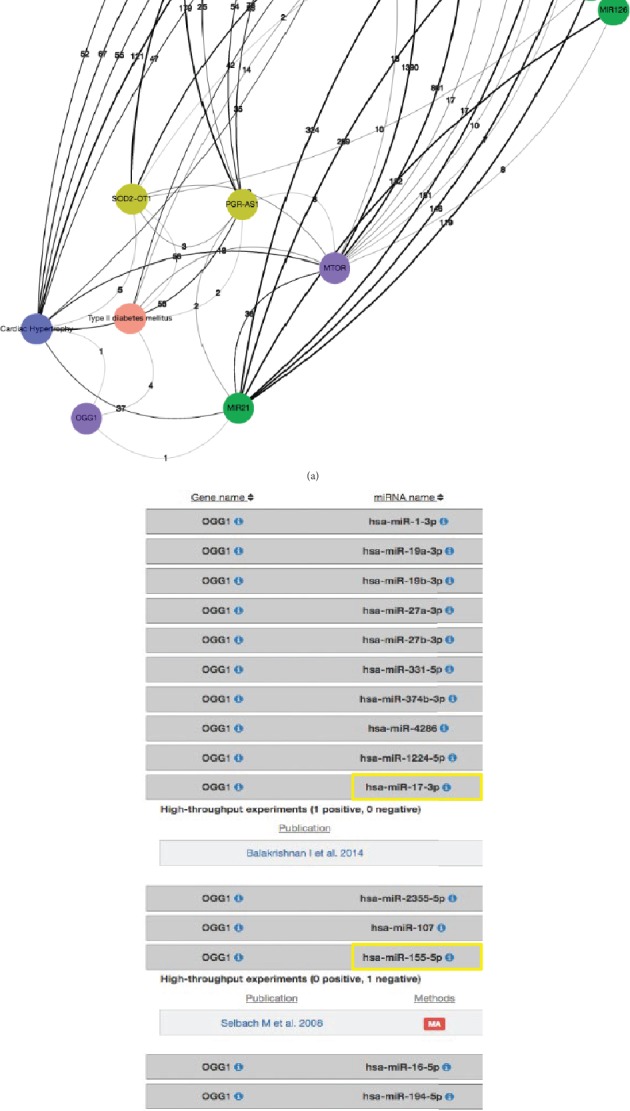
(a) An illustration of the concepts that link “Type 2 diabetes mellitus”, “Cardiac Hypertrophy”, and “OGG1”. The blue circles represent nodes from the “HFO Ontology (BioPortal) Heart Failure Ontology” dictionary, the peach circles represent the nodes from the “HP Ontology (BioPortal) Human Phenotype Ontology” dictionary, the purple circles represent the nodes from the “Human Genes and Proteins (EntrezGene)” dictionary, the greenish-yellow circles represent the nodes from the “Human Long Non-Coding RNAs, and the green circles represent the nodes from the “Human microRNAs” dictionary. The edge color is distributed across a color spectrum from black (strong association) to grey (weaker association) based on the frequency of cooccurrence. The number of publications that link the associated nodes is displayed on each edge. (b) Experimentally supported miRNA-gene interactions retrieved from the DIANA tool TarBase v.8.

Here, we attempt to search for novel insights into the association found between type 2 diabetes and cardiovascular risk, focused on oxidative stress. To do this, we start exploring DES-ROD by clicking on the “Enriched Pairs” link. In the search bars, we typed the concepts of interest “Type II diabetes” and “OGG1”, to check if this association was enriched in DES-ROD. Then, we used “OGG1” concepts' right-click menu to generate a network (Step 1). On the “Network” page, we selected the “HFO Ontology (BioPortal) Heart Failure Ontology” and the “HP Ontology (BioPortal) Human Phenotype Ontology” dictionaries; then, the “OGG1” node was highlighted and expanded with the top ten enriched associated terms from the selected dictionaries. To restrict our search to the T2DM and cardiovascular risk association, we removed all retrieved associations except “Type II diabetes mellitus” and “Cardiac Hypertrophy” (Step 2). This process was repeated by selecting the “Human Genes and Proteins (EntrezGene)”, “Human Long Non-Coding RNAs”, and “Human microRNAs” dictionaries to individually expand “OGG1”, “Type II diabetes mellitus”, and “Cardiac Hypertrophy” and then adjust the “current Threshold for pruning is: 1” (Step 3). Now, we had 4 additional nodes “MTOR”, “PGR-AS1”, “SOD2-OT1”, and “MIR21” that were similarly expanded with same dictionaries used in Step 3; then, the threshold was again adjusted “current Threshold for pruning is: 1” (see Figure 2(a), Step 4). We used the DIANA tool TarBase v.8 [118] to search if any of the miRNAs retrieved through DES-ROD target OGG1. This tool provides a collection of experimentally supported miRNA-gene interactions.  Figure 2(b) shows that this tool retrieves results for mir-155, mir-17, and mir-34, but only mir-17 interacts with OGG1.

However, Ikitimur et al. conducted a study to determine the miRNAs involved in heart failure (HF) using blood samples of 42 HF patients and 15 healthy controls [119]. They found that 29 showed miRNAs with significant dysregulation, which included upregulated miRNA-155. Moreover, miRNA-155 was positively correlated with the left ventricular mass index [119]. Marques et al. consistently demonstrated upregulated miRNA-155 in HF patients [120]. Also, He et al. confirmed the role of miRNA-155 in pathological cardiac remodeling that causes HF. They demonstrated that loss of miRNA-155 in fibroblasts protects left ventricular function after experimental acute myocardial infarction [121]. This is interesting as Corral-Fernandez et al. reported a significant correlation between the basal expression of miR-155 and miR-146a with HbA1c, glucose, and BMI [122]. This altered distribution of miR-155 and miR-146a expression related to HbA1c, glucose, and BMI was also detected using the analysis of a three-dimensional association of variables in the group of T2DM patients. Based on these findings, this group further suggested that downregulated levels of miR-155 could play an essential role in the pathogenesis of T2DM [122].

Taken together, this study demonstrates that the retrieval of miR-155 is a relevant concept to both T2DM and cardiovascular risk and serves as potentially new knowledge as to answering why T2DM and cardiovascular risk are associated.

## 6. Discussion and Limitations

DES-ROD provides users with over 10 million statistically enriched (FDR < 0.05) cooccurring concepts (with cooccurrence based on a distance up to 200 characters), compared to the documents in the background set. The cooccurring concepts or associations that are of interest to the user can be evaluated through the text from where the associations are derived; this makes it easier for users to find meaningful associations than can be used to develop novel hypotheses. However, to find meaningful associations, users should have some domain-specific knowledge. Users can also explore over 10 billion associations between any of the individual statistically enriched concepts that are semantically similar. However, this number of associations is a bit misleading; as such, associations appear to be most meaningful when the similarity between concepts is sufficiently high, i.e., >0.75.

Furthermore, DES-ROD carries all shortcomings as other text mining approaches. (1) Information extraction is restricted to electronically available documents; (2) information extraction is restricted to what the author chose to mention in the text of the manuscript, such as biomarkers, whereas the complete gene set is placed in a depository or supplementary material that DES does not analyze; (3) peer-reviewed literature contains errors that may cause literature to be omitted; (4) completeness of the concept set extracted depends on the quality and completeness of the dictionaries used and availability of synonyms of a concept; (5) some concepts are “promiscuous” and thus do not retrieve the correct information pertaining to the concept of interest; and (6) cooccurrence of terms does not necessarily imply meaningful association/link between paired terms.

Given the constraints, DES-ROD is useful as most initiated studies start with the review of literature, which DES-ROD can provide comprehensively and visually in minutes, and knowledge of this literature and summarized information extracted from it help not only with developing hypotheses but also with the interpretation of the data. Nonetheless, users should acknowledge the limitation of this system and consequently use it to draw attention to linked concepts or new emerging concepts in the field and to provide a bird's eye view on the topic of interest.

## 7. Concluding Remarks

DES-ROD rapidly and comprehensively sifts through 298,603 topic-specific publications and extracts relevant topic-specific concepts that may be known or novel. This type of information is not at all available or not easily found in other related databases. The current release comprises 131,741 statistically enriched concepts from 38 topic-relevant dictionaries, together with 10,846,802 statistically enriched pairs of concepts.

DES-ROD provides a user-friendly interface and instructional material to facilitate navigation through the KB. DES-ROD has various tools that enable users to explore enriched concepts, enriched concept pairs, or enriched associated terms based on semantic similarity between these terms, as well as the literature from which terms are derived. Users are further provided with a network viewer to visualize the associations of concepts of interests based on user-selected dictionaries, providing a flexible information exploration experience.

To our knowledge, DES-ROD is the first KB focused on RNA oxidation in human disease discoveries through literature mining and data mining. It will be updated every six months to ensure that the KB remains current. We hope that users find DES-ROD to be a useful tool for supporting RNA oxidation in human disease-related research questions.

## Figures and Tables

**Table 1:** Dictionaries used in DES-ROD with data source references.

Dictionary	Enriched unique terms in the KB	Source
Chemicals/compounds		
Chemical Entities of Biological Interest (ChEBI) [83]	19,298	Preexisting in DES
Toxins (T3DB) [84]	2,193	Preexisting in DES
Lipids (lipid maps) [85,86]	3,099	Preexisting in DES
Amyloids (Human and Mouse), compiled in-house	394	Newly compiled
Functional annotation		
Biological Process (GO) [87]	5,868	Preexisting in DES
Cellular Component (GO) [87]	1,284	Preexisting in DES
Molecular Function (GO) [87]	1,963	Preexisting in DES
Pathways (KEGG [88], Reactome [89], UniPathway [90], and PANTHER [91])	1,584	Preexisting in DES
Diseases		
DOID Ontology (BioPortal) Human Disease Ontology [92]	3,637	Preexisting in DES
ADO Ontology (BioPortal) Alzheimer's Disease Ontology [93]	937	Newly compiled
DMTO Ontology (BioPortal) Diabetes Mellitus Treatment Ontology [94]	1,980	Newly compiled
HFO Ontology (BioPortal) Heart Failure Ontology [95]	1,002	Newly compiled
CVDO Ontology (BioPortal) Cardiovascular Disease Ontology [96]	49	Newly compiled
HP Ontology (BioPortal) Human Phenotype Ontology [97]	3,306	Preexisting in DES
UBERON Ontology (BioPortal) Uber Anatomy Ontology [98]	6,657	Newly compiled
ICD9 Ontology (BioPortal) International Classification of Diseases, Version 9-Clinical Modification [99]	719	Preexisting in DES
Drugs		
Drugs (DrugBank) [100]	4,025	Preexisting in DES
ATC Ontology (BioPortal) Anatomical Therapeutic Chemical Classification [101]	2,008	Newly compiled
CSSO Ontology (BioPortal) Clinical Signs and Symptoms Ontology	206	Newly compiled
SIDER (Drug Indications and Side Effects) [102]	3,203	Preexisting in DES
Human		
Human Genes and Proteins (EntrezGene) [103]	22,896	Preexisting in DES
Human Transcription Factors [104]	1,565	Preexisting in DES
Human Transcription Cofactors (TcoF-DB) [104]	388	Preexisting in DES
Human microRNAs (HGNC [105] and EntrezGene) [106]	2,088	Updated
Human Long Noncoding RNAs (HGNC) [105]	527	Preexisting in DES
Mutations (tmVar) [107]	15,852	Preexisting in DES
Human Anatomy (in-house compiled)	2,569	Preexisting in DES
OMIT Ontology (BioPortal) Ontology for MicroRNA Target [19]	695	Newly compiled

## Data Availability

The DES-ROD portal is free for academic and nonprofit users and can be accessed at http://cbrc.kaust.edu.sa/des-rod/.

## Abbreviations

ALS: Amyotrophic lateral sclerosis
AD: Alzheimer's disease
CVD: Cardiovascular disease
FDR: False discovery rate
KB: Knowledgebase
NLP: Natural language processing
miRNA: MicroRNA
OGG1: 8-Oxoguanine glycosylase
OS: Oxidative stress
PD: Parkinson's disease
ROS: Reactive oxygen species
T2DM: Type 2 diabetes mellitus
TM: Text mining
8-oxoG: 8-Oxoguanosine.
